# Novel Insights Into Rheumatoid Arthritis Through Characterization of Concordant Changes in DNA Methylation and Gene Expression in Synovial Biopsies of Patients With Differing Numbers of Swollen Joints

**DOI:** 10.3389/fimmu.2021.651475

**Published:** 2021-04-22

**Authors:** Andrew Y. F. Li Yim, Enrico Ferrero, Klio Maratou, Huw D. Lewis, George Royal, David F. Tough, Chris Larminie, Marcel M. A. M. Mannens, Peter Henneman, Wouter J. de Jonge, Marleen G. H. van de Sande, Danielle M. Gerlag, Rab K. Prinjha, Paul P. Tak

**Affiliations:** ^1^ R&D GlaxoSmithKline, Stevenage, United Kingdom; ^2^ Department of Clinical Genetics, Genome Diagnostics Laboratory, Amsterdam Reproduction & Development, Amsterdam University Medical Centers, University of Amsterdam, Amsterdam, Netherlands; ^3^ Tytgat Institute for Liver and Intestinal Research, Amsterdam Gastroenterology & Metabolism, Amsterdam University Medical Centers, University of Amsterdam, Amsterdam, Netherlands; ^4^ Department of Surgery, University Clinic of Bonn, Bonn, Germany; ^5^ Department of Rheumatology and Clinical Immunology, Amsterdam Rheumatology and Immunology Center, Amsterdam Institute for Infection & Immunity, Amsterdam University Medical Centers, University of Amsterdam, Amsterdam, Netherlands; ^6^ Department of Experimental Immunology, Amsterdam Institute for Infection & Immunity, Amsterdam University Medical Centers, University of Amsterdam, Amsterdam, Netherlands; ^7^ Department of Rheumatology, Ghent University, Ghent, Belgium; ^8^ Department of Medicine, University of Cambridge, Cambridge, United Kingdom

**Keywords:** rheumatoid arthritis, arthralgia, DNA methylation, transcriptomics, multi-omics analyses, synovial biopsies, target identification

## Abstract

In this study, we sought to characterize synovial tissue obtained from individuals with arthralgia and disease-specific auto-antibodies and patients with established rheumatoid arthritis (RA), by applying an integrative multi-omics approach where we investigated differences at the level of DNA methylation and gene expression in relation to disease pathogenesis. We performed concurrent whole-genome bisulphite sequencing and RNA-Sequencing on synovial tissue obtained from the knee and ankle from 4 auto-antibody positive arthralgia patients and thirteen RA patients. Through multi-omics factor analysis we observed that the latent factor explaining the variance in gene expression and DNA methylation was associated with Swollen Joint Count 66 (SJC66), with patients with SJC66 of 9 or more displaying separation from the rest. Interrogating these observed differences revealed activation of the immune response as well as dysregulation of cell adhesion pathways at the level of both DNA methylation and gene expression. We observed differences for 59 genes in particular at the level of both transcript expression and DNA methylation. Our results highlight the utility of genome-wide multi-omics profiling of synovial samples for improved understanding of changes associated with disease spread in arthralgia and RA patients, and point to novel candidate targets for the treatment of the disease.

## Introduction

Rheumatoid arthritis (RA) is a complex, multifactorial, and chronic autoimmune disease that primarily affects the synovial tissue in joints (1). It affects about 1% of the population and manifests with significant unmet medical need (2). Investigating pathogenesis during different stages of disease, or across a spectrum of disease severity, is critical to optimize appropriate therapeutic interventions that affect disease progression (3, 4).

Diagnosis of established RA coincides with the development of painful and swollen joints, although circulating auto-antibodies can be detected up to 10 years before diagnosis (5, 6). Clinically manifested joint swelling reflects synovial tissue inflammation (synovitis), which is characterized by infiltration into the synovium of multiple immune cell types [including T cells, B cells, macrophages, plasma cells, dendritic cells, natural killer (NK) cells, and mast cells (7)], with up to 18 distinct infiltrating cell populations being reported on in a recent single cell transcriptomics analysis (8). As disease progresses, synovial fibroblasts adopt an increasingly aggressive and invasive phenotype, promoting further inflammation and joint damage together with other processes induced by the inflammatory environment, such as the differentiation of bone-resorbing osteoclasts (9, 10). Disease progression in early RA is often associated with the involvement of an increasing number of inflamed joints, but the mechanisms responsible for this spread of disease are poorly understood. Moreover, differences in the rate of disease manifestation and variability of response to therapy indicate that different pathophysiological mechanisms are implicated in disease development and progression compared to disease etiology (11).

An increasing body of evidence indicates that epigenetic modifications play an important role in the regulation of RA pathogenesis (12). Several array-based studies have reported widespread differences in DNA methylation among peripheral blood cells from RA patients and controls, suggesting that epigenetic modifications in circulating cells associate with disease (13, 14). However, wider conclusions may be limited by the unknown correlation of these effects to synovial cells directly at the site of inflammation. Epigenetic modifications have also been implicated in modulating the function of synovial fibroblasts in RA, through comparisons of DNA methylation patterns of cultured cells isolated from RA and osteoarthritis patients (15–20). Such studies have identified DNA methylation patterns that distinguish RA from other forms of arthritis, along with regulatory elements and biomarkers related to the pathological phenotype of RA. However, to identify novel candidate genes for therapeutic interventions that affect disease progression, it is important to study samples from patients across different stages of disease. Two such studies using cultured fibroblast-like synoviocytes found small but statistically significant patterns of hypomethylation in patients with longstanding RA compared to those with early RA, suggesting that the DNA methylome could be associated with the transformation of synovial fibroblasts into invasive cells capable of joint destruction and the resulting disease progression (21, 22).

Here, we gain insights into RA heterogeneity by combining whole-genome DNA methylome and transcriptome analyses from the same synovial biopsies and stratifying patients according to the number of swollen joints, a clinical parameter reflective of disease evolution. We find that swollen joint count based on 66 joints (SJC66), which reflects the amount and spread of inflamed synovial tissue, is associated with major changes in gene transcription and DNA methylation at promoters. By cross-interrogating differentially methylated genomic regions and their associated genes, we reveal novel candidate loci associated with the spread of the disease across joints.

## Materials and Methods

### Sample Description

Synovial tissue was collected *via* a mini-arthroscopic procedure as described previously (23) from patients at the Amsterdam University Medical Center, University of Amsterdam. A total of 17 samples were obtained from three cohorts. The first cohort contained individuals that had either arthralgia and/or a positive family history for RA, but without arthritis (as determined by a clinician), and that were positive for IgM Rheumatoid Factor (IgM-RF) and/or Anti-Citrullinated Protein Antibodies (ACPA) (Pre-synoviomics; n = 4) (24). The second cohort consisted of individuals that at inclusion were Disease Modifying Anti-Rheumatic Drug (DMARD)-naïve with early arthritis, as defined by a disease duration of less than 1 year (Synoviomics; n = 9) (25, 26). The third cohort contained samples from patients with established RA on active treatment with a disease duration of more than one year who had at least one swollen joint suitable for synovial tissue sampling (Synoviomics II; n = 4) (27). For all analyses, samples were prepared simultaneously to mitigate batch effects.

All subjects provided written informed consent and the collection and use of the samples received Institutional Review Board review and approval. Characteristics of patients included in this study are listed in **Table S1**.

### RNA-Sequencing and Whole Genome Bisulphite Sequencing

Flash-frozen synovial tissue biopsies were utilized to simultaneously isolate RNA and DNA using an AllPrep DNA/RNA Mini kit (Qiagen), with QIAshredder spin columns (Qiagen) used to disrupt the tissue. RNA samples were quantified and their integrity assessed using Qubit RNA Broad Range Assay Kit (Thermo Fisher Scientific) and an Agilent 2100 Bioanalyzer RNA 6000 Nano Kit (Agilent Technologies), respectively. Depending on sample yield, DNA samples were quantified using Qubit DNA BR or Qubit DNA HS kits (Thermo Fisher Scientific).

RNA-Seq libraries were generated from 150 ng of total RNA. The TruSeq^®^ Stranded Total RNA LT was used with a Ribo-Zero™ Human/Mouse/Rat kit (Illumina), following the ‘Low Sample’ protocol except for two modifications. Firstly, the time of the ‘Elution 2 – Frag – Prime’ program was reduced from 8 to 6 min to increase the length of the RNA fragments. Secondly, 11 instead of 15 cycles were used to enrich the DNA fragments. Libraries were quantified with a KAPA Library Quantification Kit (KAPA Biosystems) on a QuantStudio 12 K Flex Real-Time PCR System (Thermo Fisher Scientific). Five samples were multiplexed per lane and libraries were clustered and sequenced using HiSeq^®^ PE Cluster Kit v3 – cBot™ and HiSeq^®^ SBS Kit v3 kits (Illumina). Paired-end sequencing (2 x 76 bp) was performed using a HiSeq 1500 (Illumina) to a depth of ~40 M reads per sample.

Whole Genome Bisulphite Sequencing (WGBS) libraries were generated using an EpiGnome Methyl-Seq Library Preparation Kit (Epicentre, now Illumina) from 100 ng of sample DNA. Bisulphite conversion was performed using an EZ DNA Methylation-Lightning Kit (Zymo Research). Each bisulphite conversion reaction contained 500 pg of unmethylated lambda DNA (Promega), which was used as a control to verify that bisulphite conversion efficiencies were at least 98%. Libraries were quantified using a KAPA Library Quantification Kit (KAPA Biosystems) on a 7900HT Real-Time PCR System (Thermo Fisher Scientific). Six samples were multiplexed per lane and libraries were clustered and sequenced using HiSeq^®^ PE Cluster Kit v4 – cBot™ and HiSeq^®^ SBS Kit v4 kits (Illumina). Paired-end sequencing (2 x 125 bp) was performed using a HiSeq 1500 (Illumina) to a depth of ~600M reads per sample. To provide sufficient coverage each batch was sequenced over 2 high output runs.

### Exploratory Analyses of DNA Methylation and Gene Expression

To concurrently explore the DNA methylation and gene expression data, Multi-Omics Factor Analysis (MOFA) was applied (v1.0.0) (28). In short, MOFA performs unsupervised dimensionality reduction simultaneously across multiple data modalities from the same sample through a small number of inferred latent factors, enabling the detection of co-ordinated changes between the different data modalities (28). Here, we used the 5,000 most variable CpGs and genes as input and with the number of latent factors set to 9, the tolerance to 0.1 and the factor threshold to 0.02.

### Gene Expression Data Analysis

Quality assessment of the raw reads was performed using FastQC (v0.11.2) (29). Adapters were removed from reads using Scythe (v0.991) (30) and sequences were quality-trimmed using Sickle (v1.33) (31) using a quality threshold of 20. Alignment to the GRCh38 build of the human genome was performed using Kallisto (v0.44.0) (32).

All subsequent analyses were performed in R (v3.5.0) (33). Gene-level counts were generated from the transcript abundances using tximport (v1.12.0) (34). Allosome-associated genes were removed to mitigate obvious sex effects. Differential Gene Expression (DGE) analyses were conducted using DESeq2 (v1.22.2) (35) where SJC66_high_ was compared with SJC66_low_ whilst correcting for sex, age and DMARD usage using the following design formula:

$$Gene\;Expression∼sex+age+DMARD\;usage+SJC66_{dichotomized}$$

DMARD usage was a binary variable defined by the usage of any medication: conventional DMARDs (cDMARD, including methotrexate) or biological DMARDs (bDMARD). As Kallisto provided abundance levels for individual transcripts, Gene Differential Expression (GDE) analyses were also conducted to identify genes where particular transcripts were differentially expressed (36). In short, differential expression analyses was performed using DESeq2 and the resulting *p*-values were combined using the Lancaster aggregation method found in aggregation (v1.0.1) (36), where observations were weighted by the base expression. DGEs and GDEs were defined as genes with a false discovery rate (FDR)-adjusted *p*-value less than 0.05.

### DNA Methylation Data Analysis

Quality assessment of the raw reads was performed using FastQC (v0.11.2) (29). Adapter and quality trimming was performed using Skewer (v0.1.123) (37) and a quality filter of 20. To assess bisulphite conversion rates, Bismark (v0.14.1) (38) was used to align the reads to the genome of the phage lambda, and again for alignment to the GRCh38 build of the human genome. Post-alignment filtering of unmapped reads, reads aligning at multiple locations and reads with a mapping score lower than 10 was carried out using SAMtools (39).

All subsequent analyses were performed in R (v3.5.0). CpG loci located on the allosomes were removed to mitigate the sex effect. The differential methylation analyses were performed using dmrseq (v1.2.5) (40), where we contrasted SJC66_high_ with SJC66_low_ whilst correcting for sex, age and DMARD usage using the following design formula:

$$Methylation∼sex+age+DMARD\;usage+SJC66_{dichotomized}$$

Differentially Methylated Regions (DMRs) were annotated using ChipPeakAnno (v3.16.1) (41) to genes if the DMR was located within 5,000 bp upstream or 1,000 bp downstream of the gene as obtained from Gencode (v29) (42).

### Integrated DNA Methylation and Gene Expression Analysis

Integrated analyses were based on the DMRs and GDEs found through the separate DNA methylation and gene expression analyses. The overlap between DMRs and GDEs were called Genes displaying both Differential Expression and Methylation (GDEMs). For each GDEM the median percentage methylation was calculated for all constituent CpGs per sample and correlated with the log_2_ transformed expression counts to obtain the Pearson correlation coefficient. Confidence intervals (95%) were calculated through 1000 bootstraps for each GDEM. In short, 17 samples were randomly drawn from the original samples with replacement, whereupon the Pearson correlation coefficient was calculated. This process was repeated 1000 times to generate the empirical distribution function, which was then used to estimate the confidence intervals (43). The aforementioned bootstrapping approach was performed using the boot (v1.3) package (44). For inferential purposes, *p*-values were calculated by means of a permutation approach. In short, per GDEM, 1000 sets of consecutive CpGs equal to the length of the observed DMR were sampled and correlated with the gene expression signal as described above, after which the proportion of correlation coefficients higher than the observed correlation coefficient was calculated yielding the *p*-value.

### Functional Enrichment, Cell Type Enrichment and Protein-Protein Interaction Network Analyses

Gene set enrichment analyses were performed using fgsea (v1.8.0) (45) using the Metabase pathways terms (46) as reference. Metabase pathway terms with an FDR-adjusted *p*-value less than 0.05 were considered significant.

Cell proportions were imputed using xCell (v1.1.0) (47), where transcripts per million were used to estimate the proportion of each of the 64 immune and stromal cell types. Subsequent linear regressions were performed to calculate the *p*-values to assess statistical significance. Again, we compared SJC66_high_ with SJC66_low_ also correcting for age, sex and DMARD usage.

Protein-protein interaction (PPI) network analyses were performed using the STRING (v11) database (48), in order to identify whether a set of genes was over-represented for interactions. In short, the PPI analysis returned networks of genes where the encoded proteins interacted, co-expressed or co-evolved with one another, based on text mining, curated databases, and experimental data.

### Data Availability

The datasets generated and analyzed for this study can be found in the ArrayExpress repository under accession number E-MTAB-6638 and E-MTAB-6684 for WGBS and RNA-Seq, respectively. All code is hosted on GitHub at https://github.com/enricoferrero/BTCURE.

## Results

### Swollen Joint Count Is Associated With the Latent Factor Explaining Variance in Gene Expression and DNA Methylation

**Figure 1:**
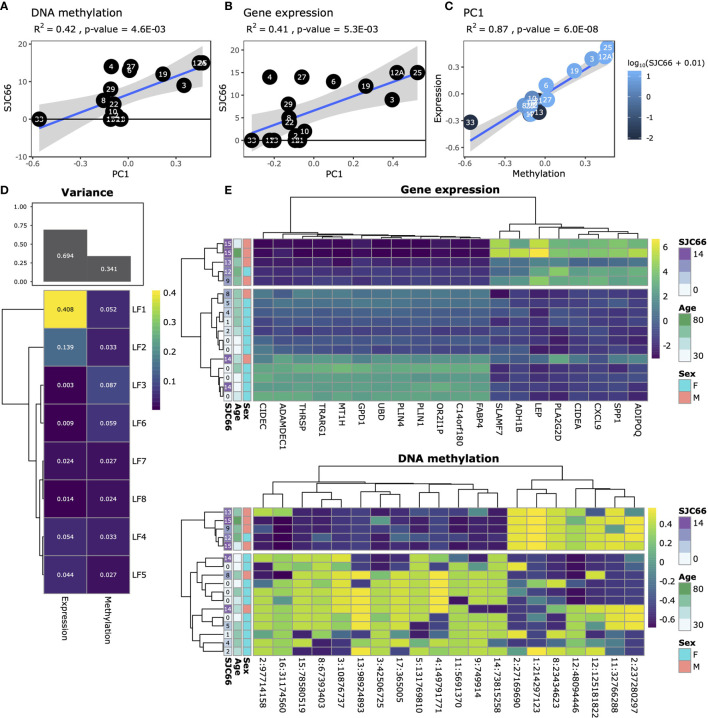
Exploratory analysis. Analysis of SJC66 regressed onto the first principal component of **(A)** DNA methylation and **(B)** gene expression annotated with the Pearson r-squared (R^2^) and the *p*-value. **(C)** Comparison of the first principal components for DNA methylation on the x-axis and gene expression together coloured for SJC66. Trendlines represent the mean and 95% confidence intervals. **(D)** Results obtained from multi-omics factor analysis, where the figure at the top depicts a bar chart representing the percentage variance explained by gene expression and DNA methylation, which has been decomposed into the separate latent factors (LF) at the bottom. **(E)** Decomposition of the variance observed for LF1 into the loadings assigned by MOFA to the top 20 weighted features for gene expression (top; colours represent log_2_(count)) and DNA methylation (bottom; colours represent percentage methylation).

We profiled the DNA methylome and transcriptome of 17 synovial tissue samples (**Table S1**) using whole genome bisulphite sequencing (WGBS) and RNA-sequencing (RNA-Seq). We initially attempted to link gene expression changes to disease duration as well as to cross-patient variations in the Disease Activity Score of 28 joints (DAS28) variables, but these analyses resulted in weak and non-biologically relevant signals, and were ultimately deemed inconclusive for this set of samples (data not shown). Principal Component Analysis (PCA) indicated that variation in both DNA methylation and gene expression were independently correlated with the swollen joint count in 66 joints (SJC66; **Figures 1A, B**). Comparison of the first principal component of both the DNA methylation and gene expression data suggested agreement with samples 33, 19, 3, 12A and 25 broadly separating from the other samples for both modalities (**Figure 1C**). While sample 33 appeared to be an outlier based on the DNA methylation data the removal thereof did not alter the correlation substantially (**Figure S1**). To further explore DNA methylation and gene expression at a genome-wide level in an integrative fashion, we performed variance decomposition using multi-omics factor analysis (MOFA) (28). MOFA infers a set of latent factors that capture sources of variability across different measured -omic modalities of the same samples. We found that most variance (approx. 70%) was better explained by gene expression as compared to DNA methylation (**Figure 1D**). Further decomposition of the variance identified 8 latent factors, with LF1 explaining 40% of the variance in gene expression, whereas variance in methylation was more evenly distributed amongst all eight latent factors. Focusing on LF1, we observed a marked separation between samples with SJC66 of 9 and above compared to those with SJC66 of 8 or less (**Figures 1E** and **S2A, B**). While most samples were obtained from the knee, two were obtained from the ankle. Interrogation of the first latent factor did not indicate any correlation with the source of the sample (**Figure S2C**). Importantly, sex was also strongly associated with LF1 (*p*-value = 8.8E-03; **Figure S2D**) where samples with SJC66 of 9 and above were mostly males and most samples with SJC66 equal to or fewer than 8 were mostly females. Therefore, subsequent comparative analyses accounted for the imbalance in sex by removal of allosome-associated genes as well as including sex as covariate in linear models. females. Therefore, subsequent comparative analyses accounted for the imbalance in sex by removal of allosome-associated genes as well as including sex as covariate in linear models.

### Differences in DNA Methylation and Gene Expression in Patients With High and Lower Numbers of Swollen Joints Are Associated With Immune Response and Cell Adhesion Pathways

**Figure 2:**
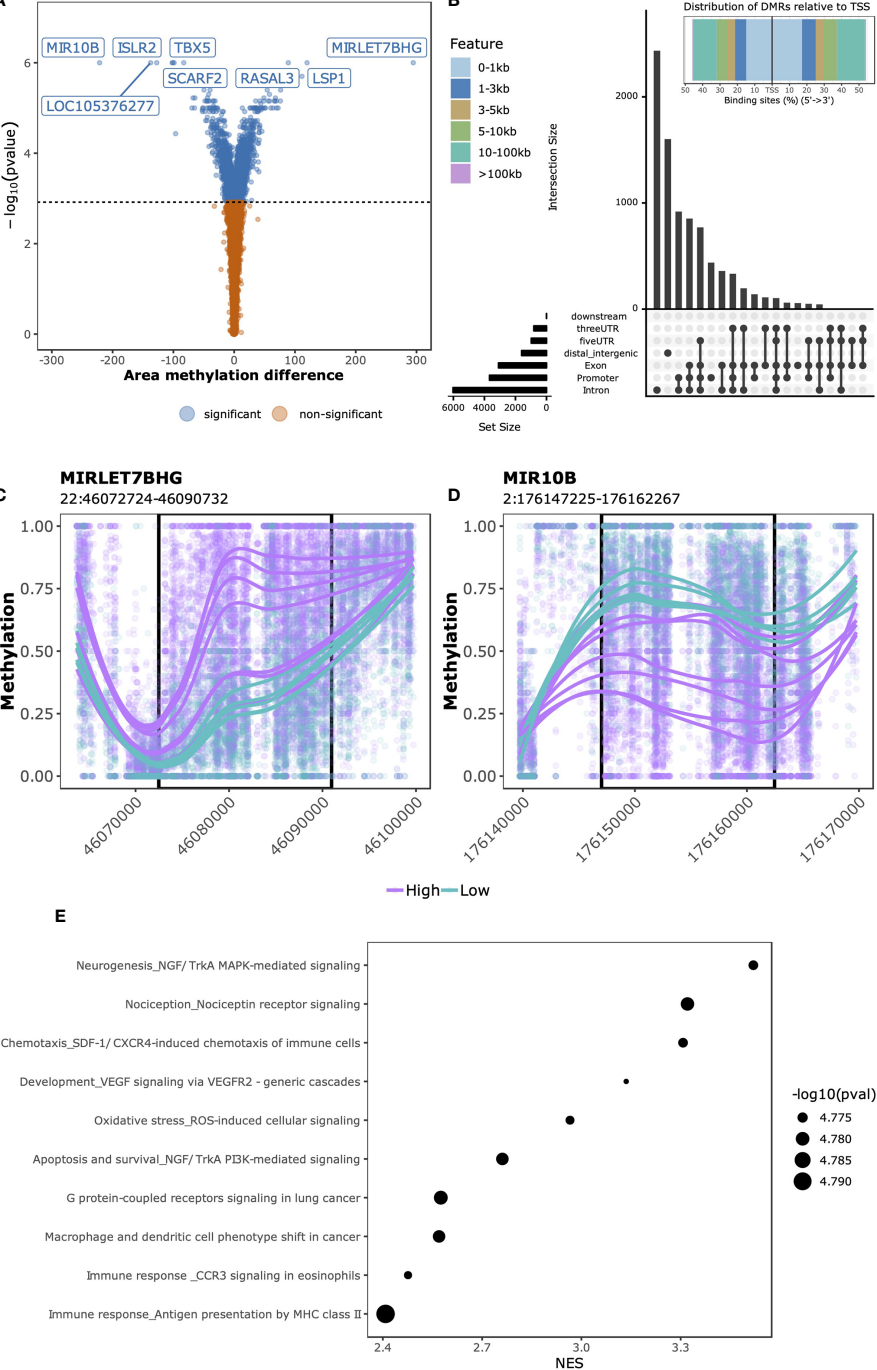
SJC66_high_ versus SJC66_low_ differential methylation analysis. **(A)** Volcano plots depicting the -log_10_(*p*-value) on the y-axis relative to the difference in % methylation when comparing the SJC66_high_ with SJC66_low_ samples on the x-axis. Colours represent the non-significant (orange) and significant regions (blue). Statistical significance is further depicted by a dashed horizontal line. **(B)** At the top: distribution of the DMRs relative to the transcription start site (TSS) and at the bottom: upset plot indicating the distribution of the DMRs across the different genetic features. **(C, D)** DMRs found at genomic co-ordinates **(C)** 22:46,072,724-46,090,732 and **(D)** 2:176,147,225-176,162,267 are located in the promoters of MIRLET7BHG and MIR10B, respectively. DNA methylation is visualized as the percentage methylation (y-axis) with a smoothed trendline per sample. **(E)** The 10 most enriched MetaCore gene sets associated to the promoter-bound DMRs. Tick marks represent gene ranks relative to the direction of the methylation effect and is summarized by the normalized expression score (NES).

Having observed genome-wide differences through exploratory analyses in both DNA methylation and gene expression data that associated with SJC66, we next investigated which regions and genes were differentially methylated and expressed. At this point we investigated DNA methylation and gene expression separately. While samples with SJC66 = 0 were included for the aforementioned exploratory analyses, they were excluded from subsequent comparative analyses as they were medically not a homogeneous group consisting of very early arthritis as well as late arthritis with no swelling following medication. The swollen samples were stratified according to the separation observed in the exploratory analyses (**Figure 1E**) and we compared SJC66_high_ (SJC66 ≥ 9) with SJC66_low_ (SJC66 < 8) correcting for age, sex and DMARD usage. Comparative methylation analysis identified 3,536 DMRs (**Figure 2A** and **Table S2**), where 2,140 were hypomethylated and 1,396 were hypermethylated. The majority of DMRs were located within 1 Kb of a TSS or in distal regions (**Figure 2B**). Notably, the most statistically significant DMRs spanned regions larger than 10 Kb (**Table S2**). For example, the top two DMRs were located within the promoter regions of the *MIRLET7B* and *MIR3619* genes (*MIRLET7BHG*; 22:46,072,724-46,090,732; **Figure 2C**) and microRNA 10B (*MIR10B*; 2:176,147,225-176,162,267; **Figure 2D**) and exceeded 15 Kb in length. While the *MIRLET7BHG*-associated DMR was hypermethylated, the *MIR10B*-associated DMR was hypomethylated when comparing SJC66_high_ with SJC66_low_. Functional analysis of the DMRs identified several pathways with evidence of differential methylation. Among the top 10, we observed that the NGF/TrkA MAPK pathway, immune response pathways including antigen presentation by MHC class II, macrophage and dendritic cell phenotype shifts, as well as CCR3 signalling in eosinophils, were hypermethylated (**Figure 2E** and **Table S3**).

**Figure 3:**
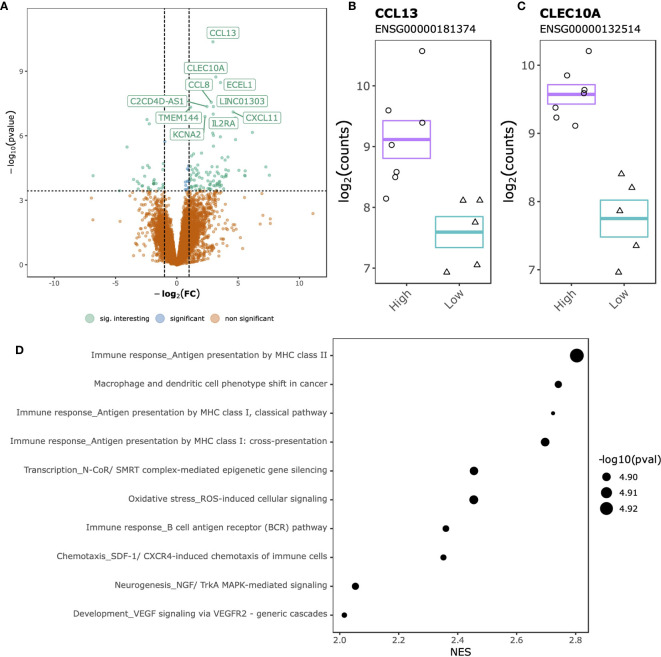
SJC66_high_ versus SJC66_low_ differential expression analysis. **(A)** Volcano plots depicting the -log_10_(*p*-value) on the y-axis relative to the difference in log_2_ fold change when comparing the SJC66_high_ with SJC66_low_ samples on the x-axis. Colours represent the non-significant genes (orange), the significant genes (blue) and the significant genes with a log_2_ fold change of larger than 1 (green; “sig. interesting”). Statistical significance is further depicted by a dashed horizontal line. Mean and standard error bars superposed onto a jitterplot for the two most differentially expressed genes **(B)** CCL13 and **(C)** CLEC10A. **(D)** The 10 most enriched MetaCore gene sets associated to the DGEs. Tick marks represent gene ranks relative to the direction of the expression effect and are summarized by the normalized expression score (NES).

Comparing SJC66_high_ with SJC66_low_ at the gene expression level identified 142 DGEs, of which 106 up-regulated and 36 down-regulated (**Figure 3A** and **Table S4**). The top 2 DGEs, chemokine ligand 13 (*CCL13*) (**Figure 3B**) and C-Type Lectin Domain Containing 10A (*CLEC10A*) (**Figure 3C**), were both found to be more highly expressed in the SJC66_high_ samples. Functional analyses revealed a striking similarity with the differential methylation results, where genes associated with antigen presentation by MHC class II as well as the NGF/TrkA MAPK pathways were overexpressed (**Figure 3D** and **Table S5**).

### Transcripts Associated With Differentially Methylated and Expressed Genes Display Concordant Expression and Identify Nodal Points for Key Interactions

**Figure 4:**
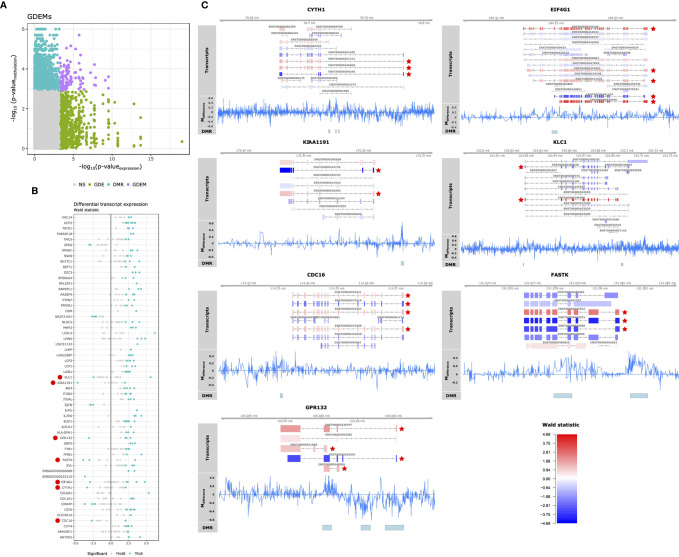
Differential methylation and alternative splicing. **(A)** Comparison of the -log_10_(*p*-values) obtained from gene differential expression analysis and differential methylation analysis on the x- and y-axis, respectively. Grey, green, blue and purple represent the genes: unchanged for methylation and gene expression (“NS”), differentially expressed (“GDE”), differentially methylated (“DMR”), differentially methylated and expressed (“GDEM”), respectively. **(B)** Visualization of the Wald statistic (a statistic calculated by DESeq2 representing the effect size relative to the variance) calculated for the individual transcripts (represented in dots) associated with each of the GDEMs. Grey and blue dots represent non-significant and significantly differentially expressed transcripts, respectively. Large red circles left of the gene symbols indicate genes with transcripts that display opposite effect sizes. **(C)** Summary visualization of the DNA methylation and gene expression signals for GDEMs: CYTH1, EIF4G11, KIAA1191, KLC1, CDC18, FASTK and GPR132. The “Transcripts” track represents individual transcripts coloured by the Wald statistic. Stars in red indicate differentially expressed transcripts. The “M_difference_” track represents the difference in percentage methylation when comparing SJC66_high_ with SJC66_low_. The “DMR” track represents the locations of the DMRs as found by dmrseq.

Having observed pathway-concordant differences in DNA methylation and gene expression, we were interested in identifying specific genes that were both differentially methylated and expressed. Although DNA methylation has traditionally been associated with gene-level expression, emerging evidence shows that it also regulates alternative splicing (49–51). Therefore, we complemented our Differential Gene Expression (DGE) analysis, which masks transcript-level dynamics, with Gene Differential Expression (GDE) analysis, which identifies genes that display transcript-level differences, by combining the *p*-values of individual transcripts associated with a single gene (36). We identified 290 genes that displayed perturbations in the expression of their transcripts (GDEs; **Table S6**). Combining the GDEs with the DMRs yielded 97 unique DMRs associated with 59 unique genes, which we termed Genes Differentially Expressed and Methylated (GDEMs) (**Figures 4A, B**). We observed that most transcripts, those associated with 52 GDEMs, were concordantly differentially expressed. Transcripts of the remaining 7 GDEMs were found to display opposite directions of expression, namely Cytohesin 1 (*CYTH1*), Eukaryotic Translation Initiation Factor 4 Gamma 1 (*EIF4G1*), a putative monooxygenase (*KIAA1191*), Kinesin Light Chain 1 (*KLC1*), Cell Division Cycle 16 (*CDC16*), Fas-Activated Serine/Threonine Kinase (*FASTK*) and G protein coupled receptor 132 (*GPR132;* **Figure 4B**). Investigation of the methylation status of the gene overlaid onto the transcript location indicated that the observed DMRs for *KIAA1191*, *KLC1*, *CDC16*, *FASTK* and *GPR132* were located in the promoter shared by most transcripts (**Figure 4C**). The mechanisms that could cause opposite direction of expression in these genes are currently unclear although studies in Arabidopsis have identified a methylation reader complex that can enhance rather than suppress gene transcription in the presence of methylation (52). Our work therefore supports the rationale for further validation and mechanistic studies.

**Figure 5:**
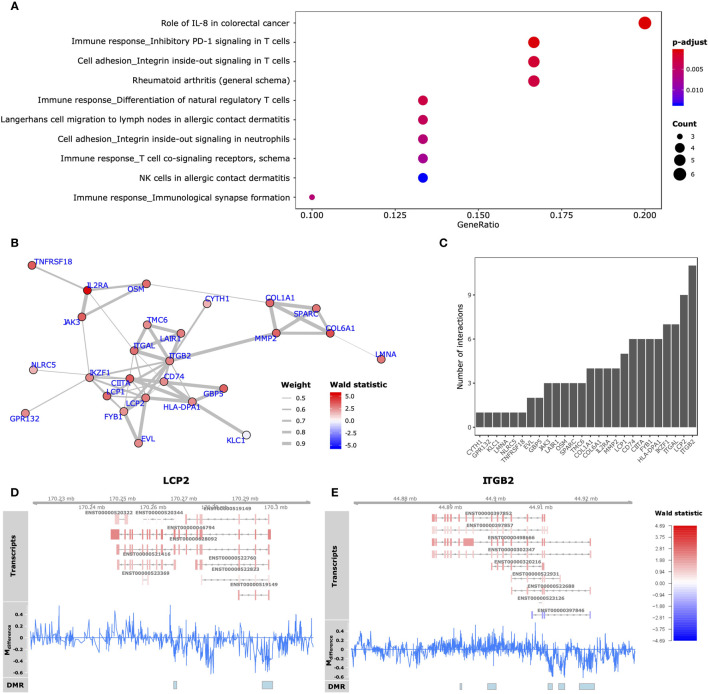
Functional analyses GDEMs. **(A)** The 10 most overrepresented MetaCore gene sets found for the GDEMs depicting the ratio of pathway-associated GDEMs relative to the total number of pathway-associated genes. The size and colour intensity are proportional to the number of pathway-associated GDEMs and the -log_10_(adjusted p-values), respectively. **(B)** GDEMs were analysed for known interactions by querying the STRING database. Represented here is the protein-protein interaction network, where the thickness of the edge is proportional to the STRINGdb evidence score and the colour proportional to the Wald statistic, respectively. **(C)** Bargraph representing the number of interactions per protein. Summary visualization of the DNA methylation and gene expression signals for GDEMs: **(D)** LCP2, **(E)** ITGB2. The “Transcripts” track represents individual transcripts coloured by the Wald statistic. The “M_difference_” track represents the difference in percentage methylation when comparing SJC66_high_ with SJC66low. The “DMR” track represents the locations of the DMRs as found by dmrseq.

Functionally, we observed that the 59 GDEMs were primarily over-represented for immune response-associated pathways, specifically T-lymphocyte associated pathways (**Figure 5A**). We interrogated which of the GDEMs encoded known interaction partners by querying the STRING database for documented interactions (48). Almost half (46%) of the GDEMs encoded for interacting proteins with the most connected GDEMs being *ITGB2* (11 interactions) and *LCP2* (9 interactions) (**Figure 5C**). Both *ITGB2* and *LCP2* were differentially methylated at multiple locations, with the largest visible differences occurring within the TSS and downstream thereof (**Figures 5D, E**). Transcript-wise, *ITGB2*-transcripts ENST00000498666 and ENST00000397852 as well as *LCP2* transcript ENST00000046794 were most differentially expressed. Expression Quantitative Trait Methylation (eQTM) analyses confirmed strong inverse correlations between the differences in methylation in the promoter regions of *ITGB2* (21:44918461-44921815) and *LCP2* (5:170295513-170298924) with transcripts ENST00000498666 (r = -0.9; *p*-value = 1E-04) and ENST00000046794 (r = -0.9; *p*-value = 1E-04), respectively (**Table 1**). While the association between the *ITGB2* promoter DMR and ENST00000397852 expression was non-significant (*p*-value = 0.2353), the correlation coefficient remained high (r = -0.87) (**Table S7**). Nonetheless, given the centrality of *ITGB2* and *LCP2* among the GDEMs would make them interesting candidates in future targeted studies.

**Table 1:** Differentially methylated and expressed genes.

Gene	GDE_p-value_	DMR	DMR_stat_	DMR_p-value_	EnsT	DTE_p-value_	eQTM_r_	eQTM_p-value_
ENSG00000233110	2.53E-04	4:185586913-185587794	-9.94	2.78E-04	ENST00000411847.1	2.53E-04	0.88 [0.74;0.95]	2.54E-02
CD74	2.00E-04	5:150410549-150413278	-9.48	3.59E-04	ENST00000009530.11	1.77E-02	-0.88 [-0.96;-0.7]	1.00E-04
CD74	2.00E-04	5:150410549-150413278	-9.48	3.59E-04	ENST00000518797.5	1.23E-02	-0.9 [-0.96;-0.76]	1.00E-04
CIITA	2.93E-10	16:10877103-10878257	-10.23	2.47E-04	ENST00000637439.1	5.06E-03	-0.92 [-0.96;-0.78]	1.00E-04
CIITA	2.93E-10	16:10877103-10878257	-10.23	2.47E-04	ENST00000637439.1	5.06E-03	-0.92 [-0.96;-0.78]	1.00E-04
CIITA	2.93E-10	16:10877103-10878257	-10.23	2.47E-04	ENST00000575513.1	8.19E-04	-0.88 [-0.96;-0.66]	8.10E-03
CIITA	2.93E-10	16:10877103-10878257	-10.23	2.47E-04	ENST00000575513.1	8.19E-04	-0.88 [-0.96;-0.66]	8.10E-03
CIITA	2.93E-10	16:10877103-10878257	-10.23	2.47E-04	ENST00000572665.1	6.67E-03	-0.86 [-0.94;-0.62]	8.80E-03
CIITA	2.93E-10	16:10877103-10878257	-10.23	2.47E-04	ENST00000572665.1	6.67E-03	-0.86 [-0.94;-0.62]	8.80E-03
CIITA	2.93E-10	16:10877103-10878257	-10.23	2.47E-04	ENST00000576601.1	3.12E-02	-0.89 [-0.96;-0.67]	2.51E-02
CIITA	2.93E-10	16:10877103-10878257	-10.23	2.47E-04	ENST00000576601.1	3.12E-02	-0.89 [-0.96;-0.67]	2.51E-02
CIITA	2.93E-10	16:10877103-10878257	-10.23	2.47E-04	ENST00000381835.9	2.59E-02	-0.86 [-0.95;-0.52]	4.06E-02
CIITA	2.93E-10	16:10877103-10878257	-10.23	2.47E-04	ENST00000381835.9	2.59E-02	-0.86 [-0.95;-0.52]	4.06E-02
CMAHP	1.89E-04	6:25232404-25233193	10.46	2.24E-04	ENST00000377993.8	2.12E-04	-0.7 [-0.87;-0.25]	1.00E-04
CMAHP	1.89E-04	6:25232404-25233193	10.46	2.24E-04	ENST00000493257.5	2.08E-02	-0.68 [-0.84;-0.28]	1.00E-04
COL6A1	1.18E-06	21:45978416-45978817	8.14	8.44E-04	ENST00000361866.7	8.83E-08	0.58 [0.07;0.8]	2.20E-03
COL6A1	1.18E-06	21:45983116-45984355	15.46	2.00E-05	ENST00000361866.7	8.83E-08	0.45 [-0.19;0.78]	1.80E-03
CYTH1	1.53E-04	17:78716750-78718002	12.83	7.40E-05	ENST00000589296.5	8.32E-04	-0.42 [-0.78;0.23]	4.12E-02
EIF4G1	4.11E-07	3:184319325-184320192	7.83	1.09E-03	ENST00000342981.8	2.97E-02	0.32 [-0.28;0.72]	1.00E-04
EIF4G1	4.11E-07	3:184319325-184320192	7.83	1.09E-03	ENST00000342981.8	2.97E-02	0.32 [-0.28;0.72]	1.00E-04
EIF4G1	4.11E-07	3:184319325-184320192	7.83	1.09E-03	ENST00000346169.6	3.07E-04	0.68 [0.28;0.9]	1.00E-04
EIF4G1	4.11E-07	3:184319325-184320192	7.83	1.09E-03	ENST00000346169.6	3.07E-04	0.68 [0.28;0.9]	1.00E-04
EIF4G1	4.11E-07	3:184319325-184320192	7.83	1.09E-03	ENST00000411531.5	1.71E-02	0.7 [0.33;0.9]	1.00E-04
EIF4G1	4.11E-07	3:184319325-184320192	7.83	1.09E-03	ENST00000411531.5	1.71E-02	0.7 [0.33;0.9]	1.00E-04
EIF4G1	4.11E-07	3:184319325-184320192	7.83	1.09E-03	ENST00000435046.6	1.78E-06	0.53 [-0.05;0.8]	1.00E-04
EIF4G1	4.11E-07	3:184319325-184320192	7.83	1.09E-03	ENST00000435046.6	1.78E-06	0.53 [-0.05;0.8]	1.00E-04
EVL	2.46E-05	14:100132409-100133139	9.08	4.52E-04	ENST00000556921.1	3.54E-02	0.89 [0.73;0.97]	3.04E-02
FASTK	1.23E-06	7:151081348-151082120	11.88	1.10E-04	ENST00000460980.5	2.32E-03	-0.21 [-0.76;0.52]	1.00E-04
FMN1	1.83E-04	15:33154437-33154950	-7.96	9.88E-04	ENST00000320930.7	2.49E-02	-0.82 [-0.94;-0.59]	1.26E-02
GPR132	3.80E-05	14:105055190-105056414	9.09	4.48E-04	ENST00000329797.7	6.12E-03	0.9 [0.62;0.99]	1.00E-04
GPR132	3.80E-05	14:105063870-105066425	-11.52	1.36E-04	ENST00000549990.1	2.60E-02	-0.38 [-0.72;0.44]	3.57E-02
IKZF1	6.58E-06	7:50305276-50312367	-11.26	1.55E-04	ENST00000471793.1	1.04E-02	-0.8 [-0.92;-0.33]	1.00E-04
IKZF1	6.58E-06	7:50305276-50312367	-11.26	1.55E-04	ENST00000492119.1	3.29E-02	-0.88 [-0.94;-0.7]	1.00E-04
IKZF1	6.58E-06	7:50305276-50312367	-11.26	1.55E-04	ENST00000413698.5	1.30E-02	-0.9 [-0.95;-0.72]	2.55E-02
IL4I1	2.32E-04	19:49895253-49897628	-7.97	9.82E-04	ENST00000391826.6	2.32E-04	-0.96 [-0.98;-0.74]	1.00E-04
ITGB2	7.75E-05	21:44898213-44900097	8.96	4.89E-04	ENST00000498666.5	5.07E-03	0.91 [0.67;0.98]	1.00E-04
ITGB2	7.75E-05	21:44911555-44912533	-11.65	1.24E-04	ENST00000397852.5	5.15E-03	-0.94 [-0.98;-0.72]	1.00E-04
ITGB2	7.75E-05	21:44911555-44912533	-11.65	1.24E-04	ENST00000498666.5	5.07E-03	-0.93 [-0.98;-0.81]	2.00E-03
ITGB2	7.75E-05	21:44911555-44912533	-11.65	1.24E-04	ENST00000522688.5	3.04E-02	-0.9 [-0.97;-0.63]	3.80E-03
ITGB2	7.75E-05	21:44911555-44912533	-11.65	1.24E-04	ENST00000320216.10	6.78E-03	-0.79 [-0.93;-0.57]	4.08E-02
ITGB2	7.75E-05	21:44911555-44912533	-11.65	1.24E-04	ENST00000302347.9	4.31E-02	-0.9 [-0.97;-0.62]	4.71E-02
ITGB2	7.75E-05	21:44913899-44915246	-12.34	9.60E-05	ENST00000397852.5	5.15E-03	-0.96 [-0.98;-0.88]	1.00E-04
ITGB2	7.75E-05	21:44913899-44915246	-12.34	9.60E-05	ENST00000397852.5	5.15E-03	-0.96 [-0.98;-0.88]	1.00E-04
ITGB2	7.75E-05	21:44913899-44915246	-12.34	9.60E-05	ENST00000498666.5	5.07E-03	-0.92 [-0.98;-0.79]	1.14E-02
ITGB2	7.75E-05	21:44913899-44915246	-12.34	9.60E-05	ENST00000498666.5	5.07E-03	-0.92 [-0.98;-0.79]	1.14E-02
ITGB2	7.75E-05	21:44913899-44915246	-12.34	9.60E-05	ENST00000302347.9	4.31E-02	-0.92 [-0.97;-0.72]	2.00E-02
ITGB2	7.75E-05	21:44913899-44915246	-12.34	9.60E-05	ENST00000302347.9	4.31E-02	-0.92 [-0.97;-0.72]	2.00E-02
ITGB2	7.75E-05	21:44913899-44915246	-12.34	9.60E-05	ENST00000522688.5	3.04E-02	-0.87 [-0.96;-0.61]	3.08E-02
ITGB2	7.75E-05	21:44913899-44915246	-12.34	9.60E-05	ENST00000522688.5	3.04E-02	-0.87 [-0.96;-0.61]	3.08E-02
ITGB2	7.75E-05	21:44913899-44915246	-12.34	9.60E-05	ENST00000320216.10	6.78E-03	-0.79 [-0.95;-0.45]	3.97E-02
ITGB2	7.75E-05	21:44913899-44915246	-12.34	9.60E-05	ENST00000320216.10	6.78E-03	-0.79 [-0.95;-0.45]	3.97E-02
ITGB2	7.75E-05	21:44918461-44921815	-18.22	9.00E-06	ENST00000498666.5	5.07E-03	-0.9 [-0.98;-0.55]	1.00E-04
ITGB2	7.75E-05	21:44918461-44921815	-18.22	9.00E-06	ENST00000498666.5	5.07E-03	-0.9 [-0.98;-0.55]	1.00E-04
ITGB2	7.75E-05	21:44918461-44921815	-18.22	9.00E-06	ENST00000320216.10	6.78E-03	-0.85 [-0.93;-0.69]	3.35E-02
ITGB2	7.75E-05	21:44918461-44921815	-18.22	9.00E-06	ENST00000320216.10	6.78E-03	-0.85 [-0.93;-0.69]	3.35E-02
KLC1	7.08E-05	14:103696092-103697307	8.07	9.05E-04	ENST00000246489.11	1.93E-08	0.62 [0.28;0.84]	2.73E-02
LAIR1	6.55E-07	19:54375122-54375966	-8.84	5.29E-04	ENST00000440716.5	9.38E-03	-0.93 [-0.98;-0.73]	3.80E-03
LCP2	1.90E-08	5:170295513-170298924	-13.19	6.10E-05	ENST00000046794.9	1.23E-03	-0.9 [-0.97;-0.66]	1.00E-04
LCP2	1.90E-08	5:170295513-170298924	-13.19	6.10E-05	ENST00000519149.1	8.96E-03	-0.82 [-0.9;-0.62]	1.00E-04
LCP2	1.90E-08	5:170295513-170298924	-13.19	6.10E-05	ENST00000628092.2	6.87E-03	-0.92 [-0.97;-0.74]	1.00E-04
LCP2	1.90E-08	5:170295513-170298924	-13.19	6.10E-05	ENST00000519594.5	2.89E-02	-0.87 [-0.93;-0.72]	3.70E-03
LCP2	1.90E-08	5:170295513-170298924	-13.19	6.10E-05	ENST00000522760.5	7.55E-03	-0.93 [-0.97;-0.8]	8.10E-03
OSM	4.92E-04	22:30266011-30268071	-13.63	5.10E-05	ENST00000215781.2	3.59E-04	-0.68 [-0.95;-0.13]	1.00E-04
OSM	4.92E-04	22:30266011-30268071	-13.63	5.10E-05	ENST00000215781.2	3.59E-04	-0.68 [-0.95;-0.13]	1.00E-04
PPP6R1	5.34E-04	19:55251330-55251959	-11.33	1.51E-04	ENST00000592242.1	4.83E-02	-0.86 [-0.94;-0.6]	4.22E-02
PPP6R1	5.34E-04	19:55251330-55251959	-11.33	1.51E-04	ENST00000412770.6	2.83E-03	-0.88 [-0.96;-0.6]	4.54E-02
RASSF4	4.87E-05	10:44977022-44977811	11	1.76E-04	ENST00000489171.5	3.42E-02	0.93 [0.76;0.98]	1.00E-04
RPS6KA4	3.11E-04	11:64359911-64360661	-10.55	2.11E-04	ENST00000334205.8	1.51E-03	-0.7 [-0.86;-0.43]	1.00E-04
RPS6KA4	3.11E-04	11:64359911-64360661	-10.55	2.11E-04	ENST00000334205.8	1.51E-03	-0.7 [-0.86;-0.43]	1.00E-04
SH3TC1	3.47E-04	4:8240533-8241022	-7.96	9.85E-04	ENST00000507891.1	6.46E-05	-0.7 [-0.83;-0.17]	1.64E-02
TMC6	6.31E-05	17:78126612-78127570	-9.6	3.35E-04	ENST00000590602.5	3.31E-02	-0.92 [-0.98;-0.82]	3.02E-02
TMC6	6.31E-05	17:78126612-78127570	-9.6	3.35E-04	ENST00000590602.5	3.31E-02	-0.92 [-0.98;-0.82]	3.02E-02
TMC6	6.31E-05	17:78126612-78127570	-9.6	3.35E-04	ENST00000591436.5	1.21E-02	-0.81 [-0.91;-0.64]	3.78E-02
TMC6	6.31E-05	17:78126612-78127570	-9.6	3.35E-04	ENST00000591436.5	1.21E-02	-0.81 [-0.91;-0.64]	3.78E-02
TMC6	6.31E-05	17:78126612-78127570	-9.6	3.35E-04	ENST00000593044.5	1.28E-02	-0.85 [-0.93;-0.67]	4.06E-02
TMC6	6.31E-05	17:78126612-78127570	-9.6	3.35E-04	ENST00000593044.5	1.28E-02	-0.85 [-0.93;-0.67]	4.06E-02
UCP2	7.66E-10	11:73979728-73982457	-9.4	3.75E-04	ENST00000545562.2	3.59E-02	-0.67 [-0.83;-0.46]	1.33E-02
UCP2	7.66E-10	11:73979728-73982457	-9.4	3.75E-04	ENST00000545562.2	3.59E-02	-0.67 [-0.83;-0.46]	1.33E-02
VAC14	2.54E-04	16:70691109-70693047	11.18	1.56E-04	ENST00000261776.9	4.82E-03	0.88 [0.55;0.96]	2.74E-02
VAC14	2.54E-04	16:70746190-70746702	9.13	4.36E-04	ENST00000261776.9	4.82E-03	0.87 [0.71;0.94]	3.48E-02

Expression quantitative trait methylation (eQTM) analysis of the GDEMs representing the correlation between methylation and transcript expression. Key in table legend: Gene = HGNC gene symbol, DMR = co-ordinates of the DMR (GRCh38), DMR_p-value_ = p-value associated to the differential methylation analysis, EnsT = Ensembl transcript ID, DTE_p-value_ = p-value associated to the differential transcript expression analysis, eQTM_r_ = Pearson correlation coefficient for the methylation-expression correlation and the 95% confidence intervals, eQTM_p-value_ = p-value associated to the methylation-expression correlation. An extended parsable table including the full eQTM analysis for all GDEMs can be found in **Table S6**.

### Estimated Cellular Composition Suggests Lower Proportion of Neuronal Cells in SJC66_high_

**Figure 6:**
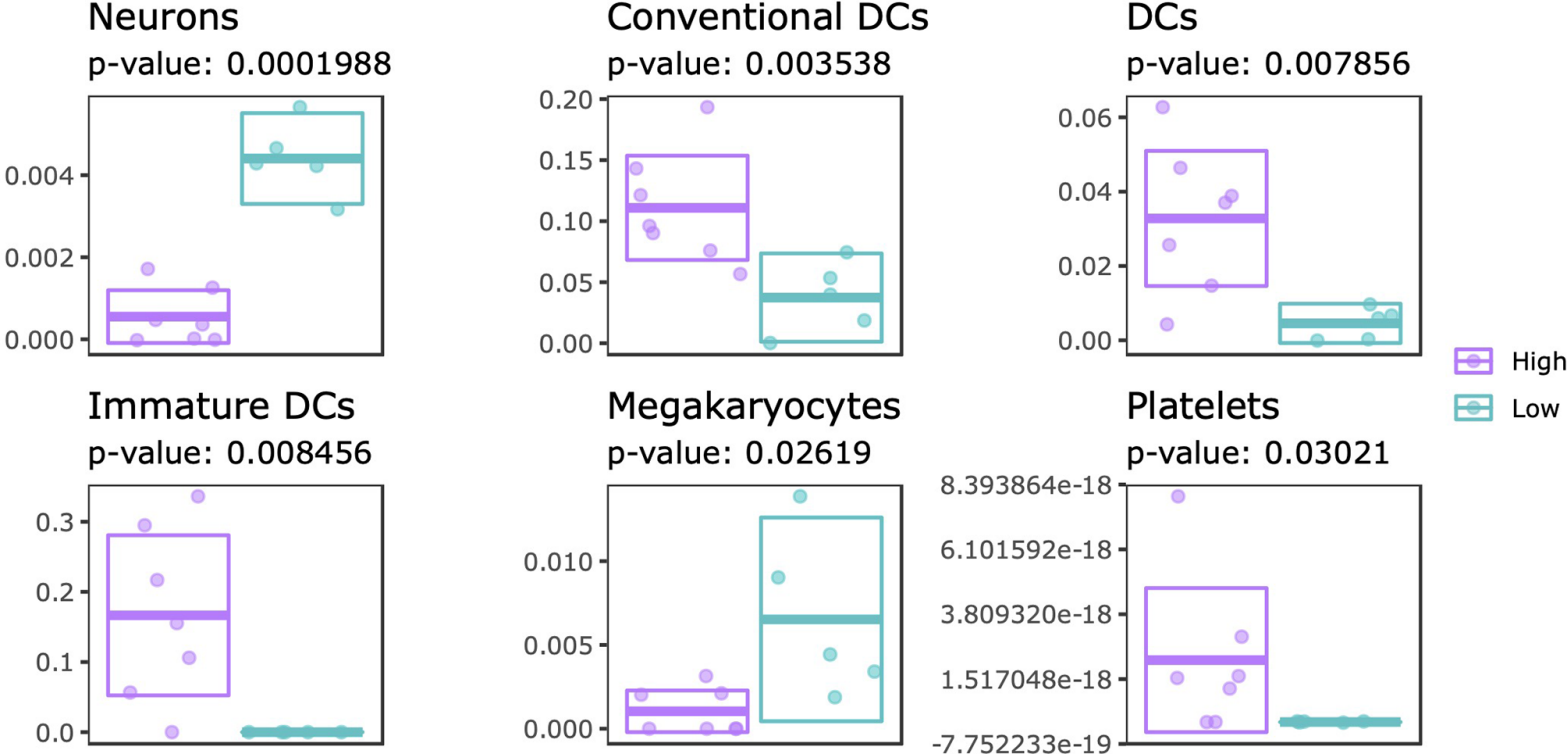
Cellular composition estimation. Cellular proportions were estimated for 64 cell types using the xCell algorithm. Of these, 6 cell types were found to be significantly different when comparing SJC66_high_ with SJC66_low_. Visualized as scatter- and cross-bar plots are the significantly differentially represented cell types, namely the neurons, conventional dendritic cells (cDC), dendritic cells (DC), immature dendritic cells (iDC), megakaryocytes, and platelets.

Systematic differences in DNA methylation and gene expression could reflect changes in the cellular composition. To this end, we estimated the cellular composition using the transcriptome data as input for xCell, which is capable of estimating enrichment scores of 64 immune and stromal cell types (47). By comparing the estimated proportions from SJC66_high_ with SJC66_low_ we identified significant differences for 6 cell types: neurons, dendritic cells (DCs: all, conventional and immature), megakaryocytes and platelets (**Figure 6** and **Table S8**). Expectedly, higher enrichment scores for DCs (all subtypes) and platelets were estimated for the SJC66_high_ samples. By contrast, lower proportions of neuron and megakaryocyte signatures were observed for the SJC66_high_ samples. Notably, the difference in neuronal enrichment was found to be the most statistically significant with an almost fourfold difference when comparing SJC66_high_ with SJC66_low_.

## Discussion

In this study, we highlight insights into RA progression by combining the outputs of parallel whole-genome DNA methylome and transcriptome analyses on extracted preparations of synovial biopsies, from auto-antibody positive individuals, early arthritis patients and patients with established RA, stratified by the number of swollen joints. We observed that synovia from patients with a higher number of swollen joints (SJC66 ≥ 9) were different at the level of DNA methylation and gene expression from synovia from patients with a lower number of swollen joints. Specifically comparing SJC66_high_ with SJC66_low_ revealed 3536 DMRs and 142 DGEs, with both datasets primarily enriched for pathways associated with immune responses. The most significant difference in methylation was found spanning the promoter regions of *MIRLET7B* and *MIR10B*. Interestingly, mouse *miR-let-7b* has been shown to provoke arthritic joint inflammation by remodeling naïve myeloid cells into M1 macrophages *via* TLR-7 ligation (53) and can augment disease severity (54). *MIR10B* has been shown to regulate Th17 cells in patients with ankylosing spondylitis (55) but no studies have specifically associated it with RA. At the level of transcription, *CCL13* and *CLEC10A* were found to be the most differentially expressed. CCL13 (MCP-4) is an extensively studied chemokine that is thought to be involved with RA pathogenesis and disease progression (56–58). By contrast, not much is known about the role of CLEC10A in RA besides it being highly expressed on immature dendritic cells (DCs), monocyte-derived DCs and alternatively activated macrophages (59), as well as having been observed in the inflamed synovium of patients with active RA (60). Chemokines CCL13, CCL8, CXCL11, CXCL10, and CXCL9 regulate the recruitment of leukocytes into tissue and have therefore been implicated in the pathogenesis of RA (61). Differential methylation was observed in the vicinity of the promoter for *CCL13*, *CXCL11*, and *CXCL9*. Such results support a role for epigenetic/transcriptional processes in the spread of pathology to additional joints. While definitive mechanisms of joint spreading remain elusive, possible roles for immune cell migration due to chemokine expression (62, 63) could be further evaluated based on our data.

Altogether, we observed that 3% of DMRs associate with 20% of the differentially expressed GDEs. It is not surprising that not all DMRs could be linked to genes as a large number are found in distal intergenic (18.6%) or intronic (28.3%) regions, making any functional inference challenging. Of the genes that presented both differentially expression and methylation, protein-protein interaction networks indicated that half encoded for interacting proteins, suggesting that the observed GDEMs function together. The most interconnected GDEMs appeared to be *ITGB2* and *LCP2*, with multiple regions of differential methylation observed surrounding both genes. While we observed transcript differential expression, most transcripts belonging to *ITGB2* and *LCP2* behaved similarly and all displayed reasonably strong inverse correlations with the DMRs located in the promoter area. *ITGB2* encodes an integrin, which would typically be involved in cell-surface mediated signalling. We observed that the gene encoding ITGB2’s interaction partner integrin alpha L (*ITGAL*) was also differentially methylated and expressed. ITGB2 and ITGAL together form lymphocyte function-associated antigen (LFA-1), which interacts with intracellular adhesion molecule 1 (ICAM-1 or CD54) resulting in an enhanced immune cell influx into the synovial tissue (64, 65). Inhibiting LFA-1 has been reported to reduce inflammation and joint destruction in murine models of arthritis (66).

Functionally, the 59 GDEMs were primarily over-represented for immune response-associated pathways, specifically T-lymphocyte associated ones. It would be fascinating to understand why the DNA methylome and transcriptome between patients with a SJC66 of 9 and above relative to patients with a SJC66 of 8 present with such a sudden split instead of a gradual difference. It is clear that inflammation is likely an important factor contributing to the observed differences as patients with a high SJC66 also generally present higher levels of inflammation as expressed through using erythrocyte sedimentation rate (ESR) or concentration C-reactive protein (CRP). Our transcriptomic data indeed suggested an increased proportion of immune cells among SJC66_high_ samples, as would be expected while pathology develops and cells migrate into the affected joints. Previous work has shown that clinically manifest arthritis in established RA is associated with increased infiltration of leukocytes. In synovial tissue samples from clinically involved joints, scores for infiltration by DCs are consistently higher than in clinically uninvolved joints obtained simultaneously from the same RA patients (67). Importantly, when comparing different clinically inflamed joints from the same RA patient simultaneously, leukocyte infiltration in one inflamed joint was shown to be representative of that in other inflamed joints, supporting the notion that leukocytes migrate from one joint to another (68). Indeed, there is continuous influx of leukocytes into the joints in established RA (69). We postulate that if synovial leukocytes exhibit properties that would facilitate cell migration, arthritis might spread from one inflamed joint to another. The results presented here support a disease mechanism in which, after development of clinically established RA, inflammatory and cell adhesion-associated processes play a key role in the progression of RA to greater joint involvement (70–72). Interestingly, we also observed differences of neuronal signatures suggesting a lower relative enrichment of neurons among SJC66_high_ samples. In addition, enrichment analyses on the DMRs suggested hypermethylation of genes encoding nociception receptors, which are typically associated with peripheral sensory neurons (73, 74). A similar decrease in neuronal signature has previously been associated with RA severity, where the authors noted a potential role in the maladaptive response towards damage (75). This is consistent with a more general loss of anti-inflammatory control by the nervous system in RA (76). Important to note is that our observations are based on estimates made by xCell, which can only calculate enrichment scores based on signatures rather than absolute values of cells. We are therefore unable to discern whether a population increased in size or a different population had decreased. Ideally, a similar estimate would have been generated based on the DNA methylation data, but the currently available reference datasets do not include the cell types measured available in xCell.

There are two limitations of this study, namely the fact that sex confounds the separation between SJC66_high_ and SJC66_low_ and the limited sample size. While we have sought to mitigate the confounding effect of sex by removing genes and CpGs located on the allosomes as well as by including sex as a covariate in our analyses, we acknowledge that we cannot fully eliminate the possibility that a sex effect is present. Accordingly, validation studies would be necessary where the DMEGs are verified using a larger, independent cohort while controlling for an interaction effect between sex and SJC66. The observed differences in transcript expression could be validated using a quantitative PCR approach with primers designed specifically against particular transcripts. Similarly, for validating the DMRs, targeted bisulphite-sequencing using primers for the regions of interest would be a cost-effective approach.

In conclusion, our study constitutes an exploratory analysis of whole genome DNA methylation and gene expression data performed on primary synovial tissue material from auto-antibody positive arthralgia patients without arthritis as well as patients with early and established RA patients. Where previous studies investigated cells from patients with RA versus disease controls and were potentially limited by their use of cultured cells, we focus on an integrative analysis of epigenetic marks and alternative splicing associated with swelling spread, providing novel insights into the mechanisms of disease progression towards more severe phenotypes. Nonetheless, further validation is necessary if the identified target genes are to be used for monitoring or treatment of the swelling and associated inflammatory processes in the joints of RA patients.

## Data Availability Statement

The datasets presented in this study can be found in online repositories. The names of the repository/repositories and accession number(s) can be found below: https://www.ebi.ac.uk/arrayexpress/, E-MTAB-6638; https://www.ebi.ac.uk/arrayexpress/, E-MTAB-6684.

## Ethics Statement

The studies involving human participants were reviewed and approved by AMC medisch ethisch toetsingscommissie under MEC 02/152, MEC 05/107, and MEC 07/253 for the Synoviomics, Pre-synoviomics, and Synoviomics II cohorts, respectively. Written informed consent was obtained from the individual(s) for the publication of any potentially identifiable images or data included in this article.

## Author Contributions

EF, HL, DT, CL, MS, DG, PT, and RP conceived the study. KM and GR carried out the laboratory experimental work. EF and CL conceived the analytical design. EF and AL performed the data analysis. HL, DT, CL, MM, PH, and WJ helped supervise the secondary data analyses. EF, AL, KM, HL, and DT led the writing of the manuscript. All authors contributed to the article and approved the submitted version.

## Funding

AL was funded by the European Union’s Horizon 2020 research and innovation program under Grant Agreement No. ITN-2014-EID-641665. This study was supported by the Dutch Arthritis Foundation (06-1-303 and 11-1-407), IMI BeTheCure (115142), and FP7 Euro-TEAM consortium (305549).

## Conflict of Interest

AL, KM, GR, HL, DT, CL, DG, and RP were employed by GSK when this study was conducted. EF and PT were employed by Novartis and Candel Therapeutics when this study was conducted, respectively. WJ was financially supported by GSK and Mead Johnson.

The remaining authors declare that the research was conducted in the absence of any commercial or financial relationships that could be construed as a potential conflict of interest.
