# Supplementary material for: Novel Insights Into Rheumatoid Arthritis Through Characterization of Concordant Changes in DNA Methylation and Gene Expression in Synovial Biopsies of Patients With Differing Numbers of Swollen Joints
Source: Front Immunol. 2021 Apr 22;12:651475. doi: 10.3389/fimmu.2021.651475 (PMC8100206; doi:10.3389/fimmu.2021.651475)
Supplement: Supplementary file 1 [file DataSheet_1.docx]

Supplementary Material

# Supplementary Figures and Tables

## Supplementary Tables

**Table S1. Patient characteristics.** Biological characteristics associated rheumatoid arthritis for each sample. RF = Rheumatoid Factor, ACPA = Anti-Citrullinated Protein Antibody, CCP = Cyclic Citrullinated Protein, ESR = Erythrocyte Sedimentation Rate, CRP = C-reactive protein, DAS28 = Disease Activity Score 28, VAS = Visual Analogue Scale, TJC68 = Tender Joint Count 68, SJC66 = Swollen Joint Count 66. Joint = The original joint from which the biopsy was obtained. DMARD usage = Whether the patient from which the sample was taken was on DMARD treatment.

**Table S2. Differentially methylated region (DMR) analysis comparing samples SJC66_high_ with SJC66_low_.** Key in table legend: chr = chromosome of the DMR, start = start of the DMR, end = end of the DMR, width = width of the DMR, L = number of CpGs in DMRs, area = methylation area of the DMR as measure of degree of methylation, beta = difference in methylation, stat = Stouffer statistic, pval = *p*-value, qval = q*-*value, insideFeature = feature hosting the DMR, distanceToSite = distance to the nearest feature, ENS = Ensembl gene ID, hgnc = HGNC gene symbol, entrez = Entrez gene ID.

**Table S3. Gene set enrichment analysis results of the differentially methylated regions when comparing SJC66_high_ versus SJC66_low_.** Key in table legend: Pathway = MetaCore pathway, pval = *p*-value, padj = FDR adjusted *p-*value, ES = Enrichment score, NES = Normalized enrichment score, nMoreExtreme = number of times a random gene set had a more extreme enrichment score, size = Size of the gene sets.

**Table S4. Differential gene expression analysis (DGE) comparing samples SJC66_high_ with SJC66_low_.** Key in table legend: ENS = Ensembl gene ID, baseMean = Mean gene expression level across all samples, log2FoldChange = log_2_ fold change, lfcSE = log_2_ fold change standard error, stat = Wald statistic, pvalue = *p*-value, padj = FDR adjusted *p-*value, chr = chromosome of the gene, hgnc = HGNC gene symbol, entrez = Entrez gene ID.

**Table S5. Gene set enrichment analysis results of the differential gene expression analysis when comparing SJC66_high_ versus SJC66_low_.** Key in table legend: Pathway = MetaCore pathway, pval = *p*-value, padj = FDR adjusted *p-*value, ES = Enrichment score, NES = Normalized enrichment score, nMoreExtreme = number of times a random gene set had a more extreme enrichment score, size = Size of the gene sets.

**Table S6. Gene differential analysis (GDE) comparing samples SJC66_high_ with SJC66_low_.** Key in table legend: ENS = Ensembl Gene ID, n = number of measured transcripts associated to that gene, pvalue_lanc = Lancaster aggregated *p*-value, padj_lanc = FDR-adjusted *p*-value, chr = chromosome of the gene, hgnc = HGNC gene symbol.

**Table S7. Genes that are differentially expressed and methylated when comparing samples SJC66_high_ with SJC66_low_.** Key in table legend: ENSG = Ensembl Gene ID, ENST = Ensembl Transcript ID, log2FC = log_2_ fold change, dte_wald_stat = Wald statistic associated to the differential transcript expression analysis, dte_pvalue = *p*-value associated to the differential transcript expression analysis, dte_padj = FDR-adjusted *p*-value associated to the differential transcript expression analysis, hgnc = HGNC gene symbol, entrez = Entrez gene ID, dmr_chr = chromosome of the DMR, dmr_start = start of the DMR (GRCh38), dmr_end = end of the DMR (GRCh38), dmr_width = width of the DMR in nucleotides, dmr_area = area of the DMR, dmr_stat = test statistic of the DMR, dmr_pvalue = *p*-value of the DMR, dmr_qvalue = *q*-value of the DMR, dmr_distance_feature = distance of the DMR to the nearest promoter, dmr_feature = genetic feature containing the DMR, tx_n = number of transcripts associated to the gene, gde_pvalue = *p­*-value of the gene differential expression analysis (i.e. Lancaster aggregated *p*-value), gde_padj = FDR-adjusted *p­*-value of the gene differential expression analysis, eqtm_corcoef = Pearson correlation coefficient obtained from the eQTM analysis, eqtm_corcoef_ci95_lower = lower bound of the 95% confidence interval, eqtm_corcoef_ci95_upper = upper bound of the 95% confidence interval, dmr_ncpgs = number of CpGs located in the DMR, eqtm_pval = *p*-value of the correlation coefficient.

**Table S8. Estimated cellular composition.** Linear regression analyses to evaluate the difference between the estimated cellular proportions as calculated using xCell. Key in table legend: Mean SJC66_low_ = Mean proportion SJC66_low_, Mean SJC66_high_ = Mean proportion SJC66_high_, Mean difference = Mean difference between SJC66_high_ and SJC66_low_. T-statistic = T-statistic associated with the mean difference. *p*-value = *p*-value associated to the mean difference.

## Supplementary Figures

**Figure S1. Correlation analyses of principal component 1 with SJC66 in the absence of sample 33.** Analysis of SJC66 regressed onto the first principal component of (a) DNA methylation and (b) gene expression annotated with the Pearson r-squared (R^2^) and the *p*-value. Coloured for SJC66. Trendlines represent the mean and 95% confidence intervals.

**Figure S2. Correlation analyses of latent factor 1 with different metadata.** Ordinary linear regressions of latent factor 1 as calculated using multi-omics factor analysis regressed against (a) SJC66, (b) SJCC66 dichotomized at 8, (c) joint and (d) sex from which the sample was derived. Each analysis was annotated with p-value, with the regression against SJC66 including R^2^.
