# Supplementary figures and images for: Novel Insights Into Rheumatoid Arthritis Through Characterization of Concordant Changes in DNA Methylation and Gene Expression in Synovial Biopsies of Patients With Differing Numbers of Swollen Joints
Source: Front Immunol. 2021 Apr 22;12:651475. doi: 10.3389/fimmu.2021.651475 (PMC8100206; doi:10.3389/fimmu.2021.651475)

**a** DNA methylation (no sample 33)  
 $R^2 = 0.4$  , p-value = 8.4E-03

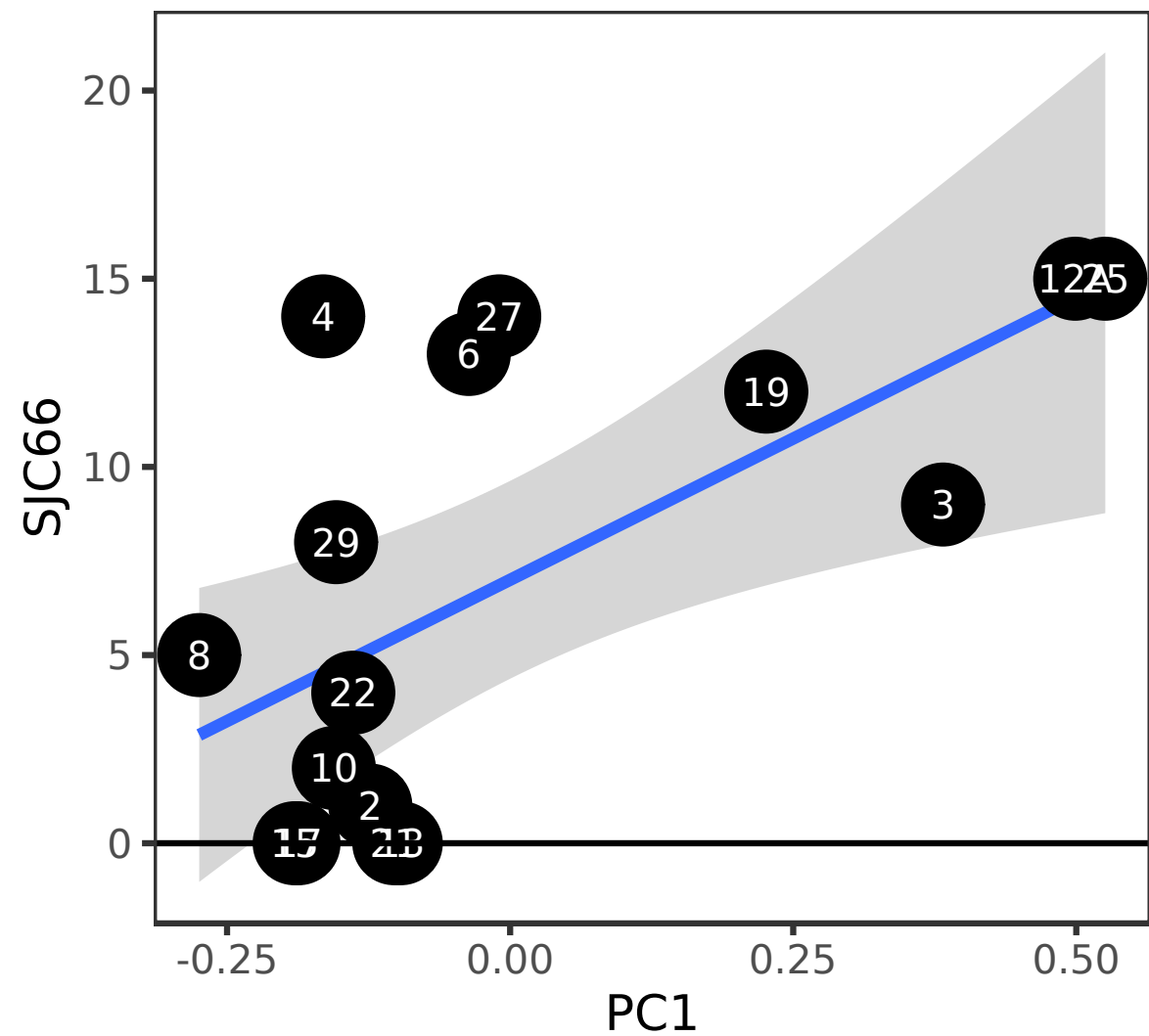

**b** Gene expression (no sample 33)  
 $R^2 = 0.4$  , p-value = 8.4E-03

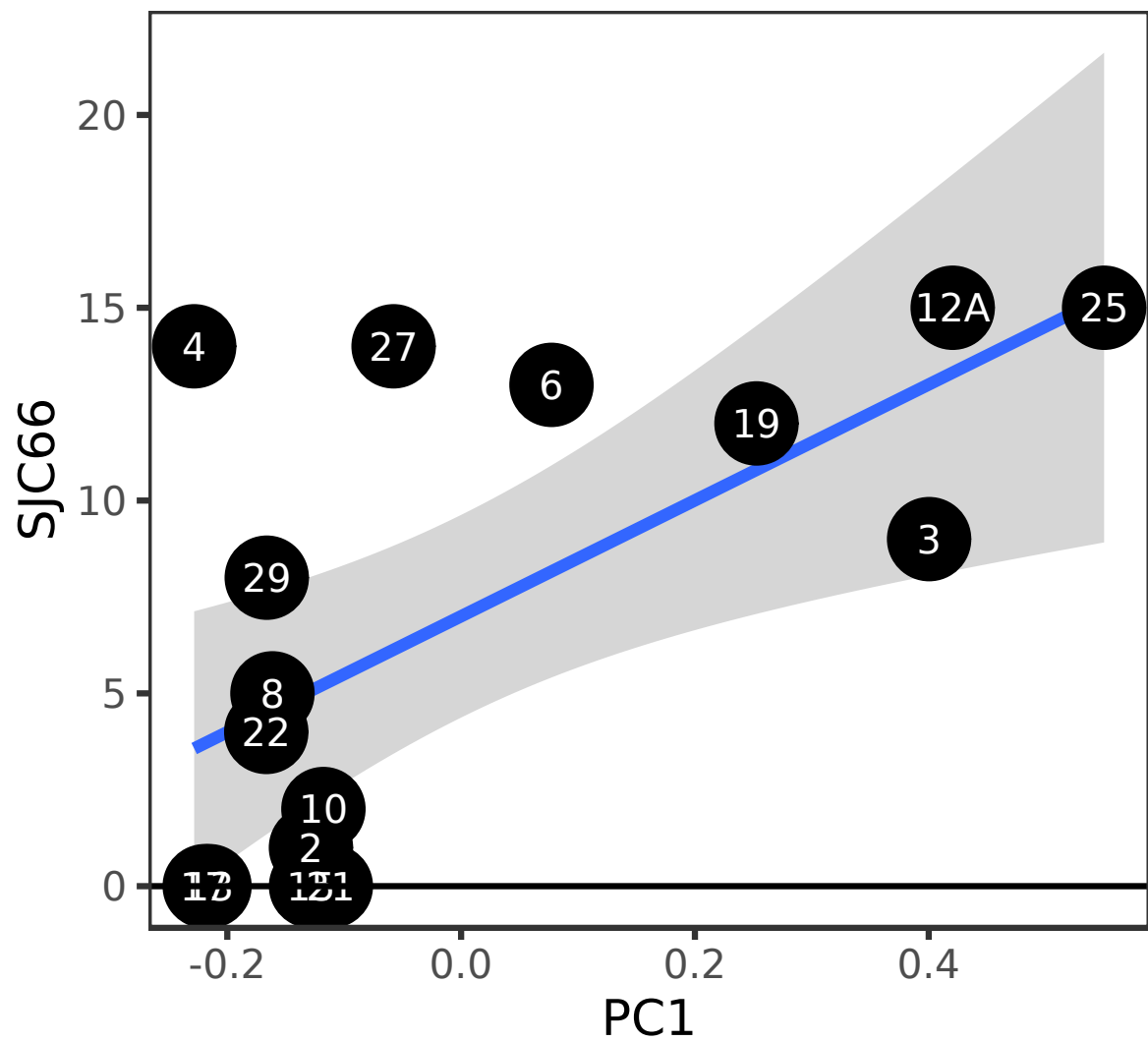

Supplement: Supplementary file 2 [file Image_1.pdf]

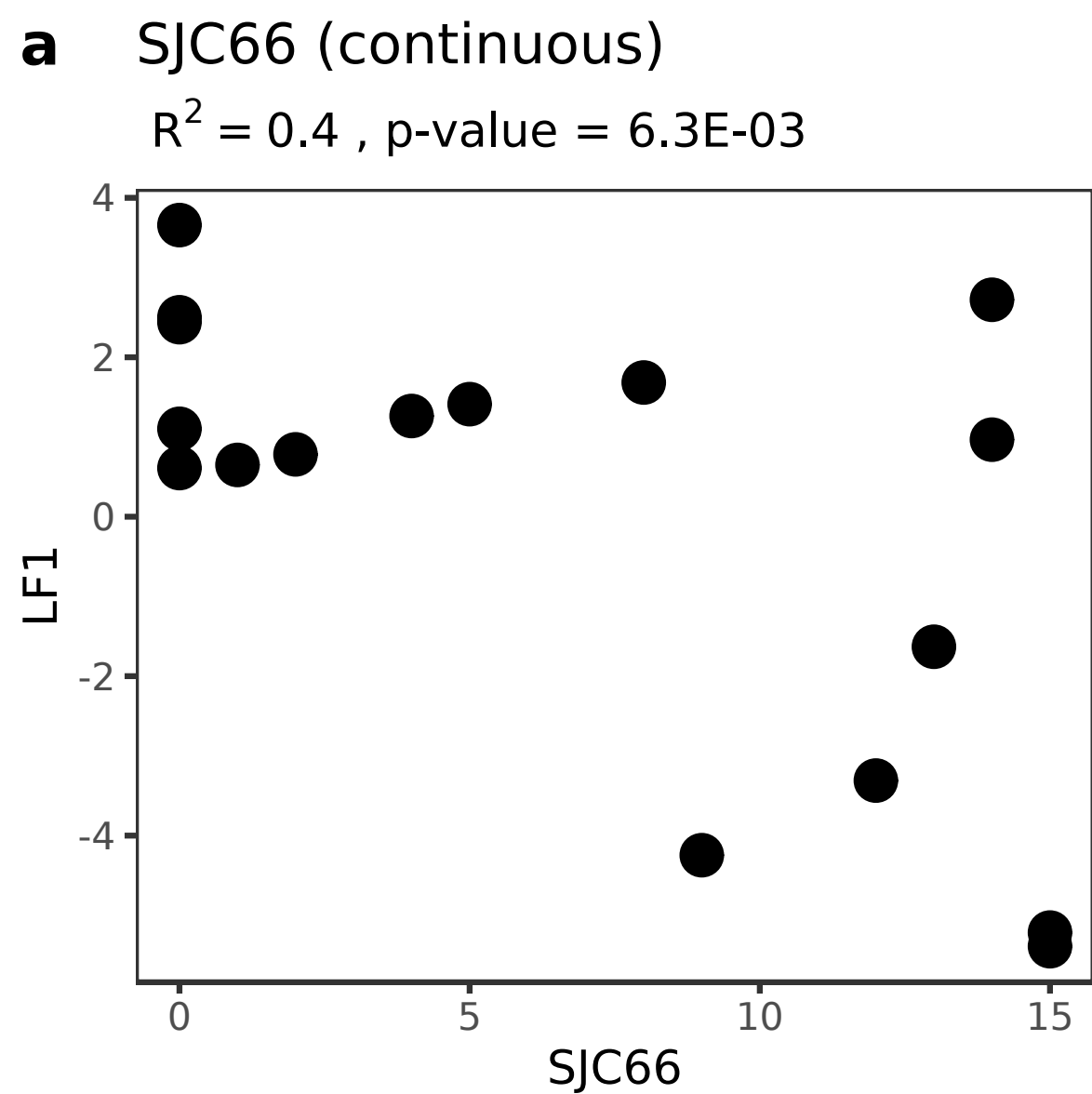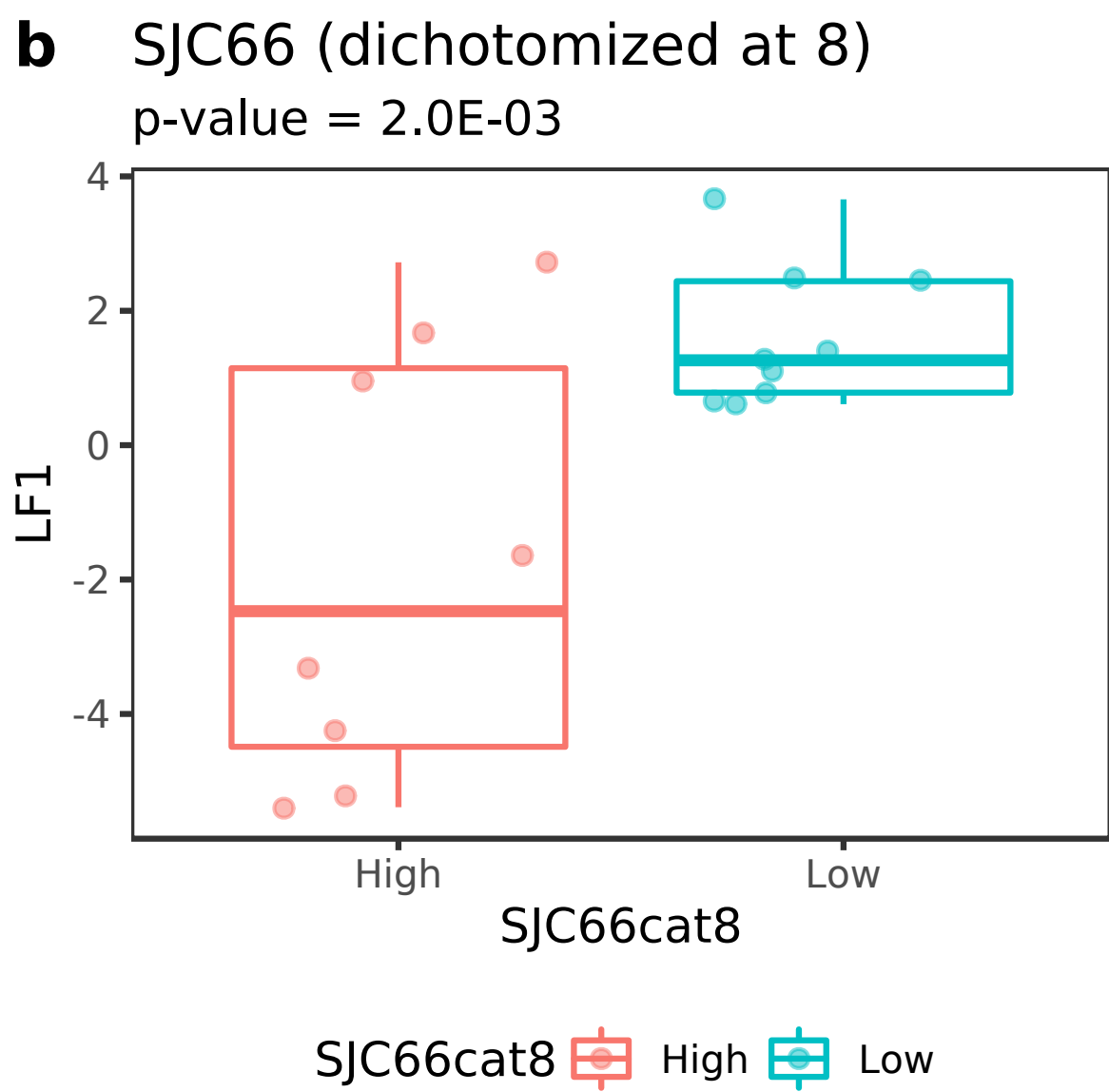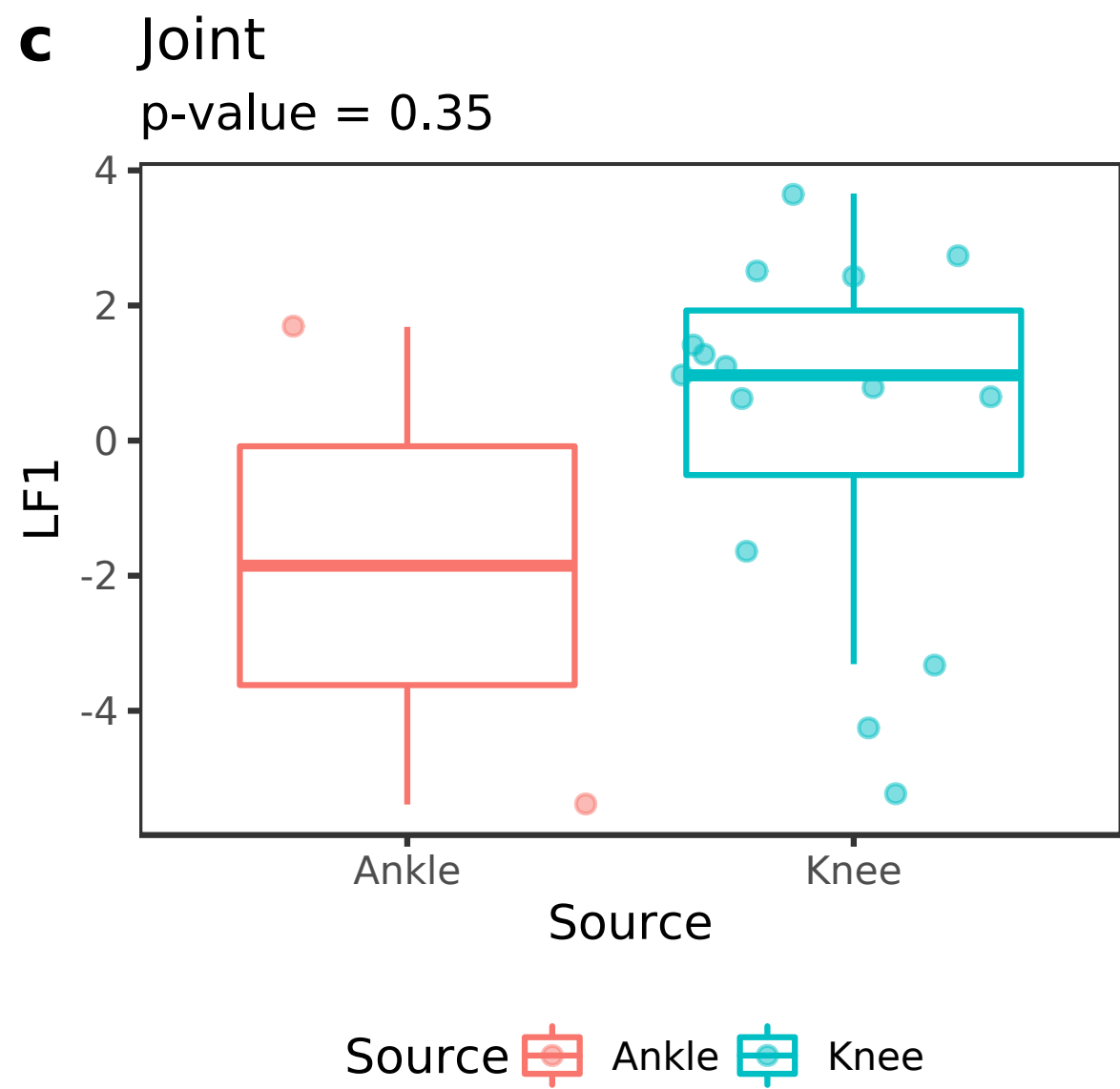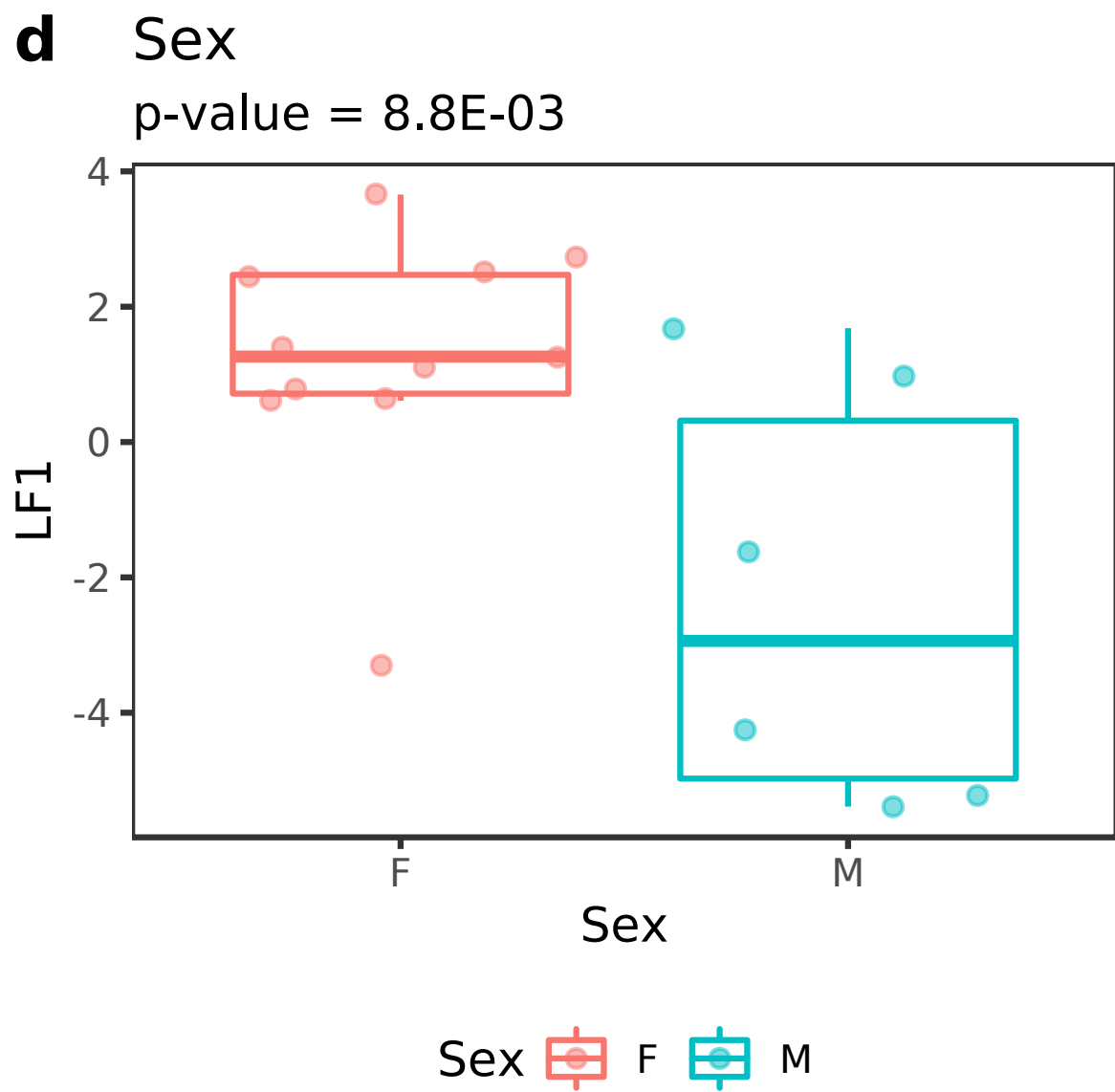

Supplement: Supplementary file 3 [file Image_2.pdf]
